# Delayed Diagnosis of Iatrogenic Bladder Perforation in a Neonate

**DOI:** 10.1155/2016/1425373

**Published:** 2016-09-26

**Authors:** Antoinette S. Birs, Jose A. Perez, Mark A. Rich, Hubert S. Swana

**Affiliations:** ^1^College of Medicine, University of Central Florida, 6850 Lake Nona Blvd, Orlando, FL 32827, USA; ^2^Neonatology, Winnie Palmer Hospital, 83 W. Miller St, Orlando, FL 32806, USA; ^3^Pediatric Urology, Arnold Palmer Children's Hospital, 92 W. Miller St, Orlando, FL 32806, USA

## Abstract

Iatrogenic bladder injuries have been reported in the neonate during umbilical artery/vein catheterization, voiding cystourethrogram, urinary catheterizations, and overwhelming hypoxic conditions. Patients with iatrogenic bladder perforations can present with acute abdomen indicating urinary peritonitis, septic-uremic shock, or subtle symptoms like abdominal distension, pain, hematuria, uremia, electrolyte imbalances, and/or difficulty urinating. The following neonatal case report of perforated bladder includes a review of the signs, symptoms, diagnostic tools, and management of bladder injury in neonates.

## 1. Introduction

Iatrogenic bladder perforations in the pediatric population reported in the literature typically result from complications during inguinal hernia repairs, voiding cystourethrograms, or bladder or umbilical catheterizations [1].

Predisposing factors such as hypotonia as seen in Down syndrome, connective tissue disorders as in Ehlers-Danlos syndrome, or structural malformations such as posterior urethral valves should alert urologists to take special precautions with these patients because they may be at a higher risk of iatrogenic injury. The infant anatomy itself contributes to an increased risk of iatrogenic perforation. The bladder lies in a higher region of the abdomen and the bladder walls are thinnest during infancy, making them perceptible to iatrogenic rupture.

Several reports of bladder ruptures secondary to profound hypoxic states have been reported [1, 2]. It is of interest to determine what role the hypoxic state plays in patients with iatrogenic bladder ruptures and if all neonates experiencing a large degree of hypoxia should be placed on a specific protocol to watch for spontaneous or iatrogenic ruptures.

## 2. Case Presentation

**Figure 1:**
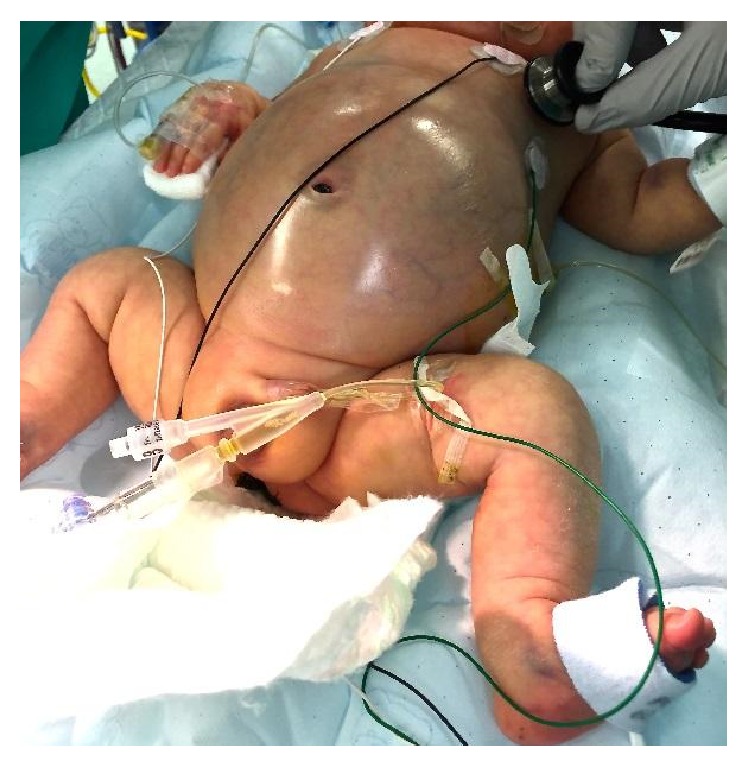
Massive abdominal ascites in neonate prior to exploratory laparotomy.

**Figure 2:**
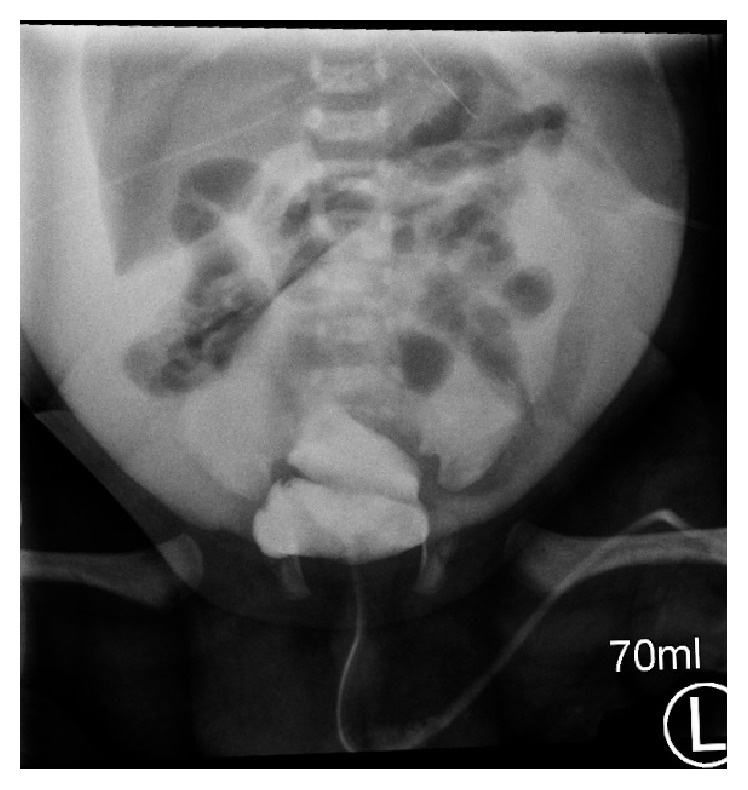
Cystourethrogram indicating intraperitoneal bladder rupture.

We describe a 29-day-old infant male who was born at 37 weeks' gestation to a 29-year-old G1P0. The patient weighed 3528 grams at birth and APGAR scores were 1, 3, and 6 at 1, 5, and 10 minutes. The patient was transferred to the NICU after experiencing seizure-like activity and respiratory distress. Blood cultures grew* E. coli*. The patient continued to deteriorate with evidence of persistent pulmonary hypertension of the newborn (PPHN). Inhaled nitric oxide, high frequency oscillatory ventilation, and epinephrine drip therapy were initiated. Overall, the patient did not respond and was placed on extracorporeal membrane oxygenation (ECMO) on day of life (DoL) number 5. As per protocol, a Foley catheter was placed at this time without difficulty. Based on abnormal renal function, a renal ultrasound was performed the day after ECMO canalization. It revealed moderate right and mild to moderate left renal pelvicaliectasis and bilateral echogenic appearance of the kidneys as well as a small volume abdominal ascites. On day of life number 11, the patient was taken off ECMO. Over the course of the next week, increasing abdominal distension and an inability to void without a Foley catheter prompted an abdominal ultrasound. Increased ascites and diffuse increased echogenicity consistent with marked diffuse renal parenchymal abnormality were reported. On day of life 25, a peritoneal tap analysis revealed high level of red blood cells (493/L), creatinine (53 *μ*mol/L), lactate dehydrogenase (276 U/L), and triglycerides (32 mg/dL). On day of life 29, the urology team was consulted for persistent and worsening abdominal ascites and inability to wean off the Foley catheter (Figure 1). The serum, peritoneal, and urinary creatinine levels were 39 *μ*mol/L, 53 *μ*mol/L, and 442 *μ*mol/L, respectively. On hospital day 31, a voiding urethrocystogram was performed at the bedside. It revealed an intraperitoneal leak secondary to a bladder rupture (Figure 2). The patient underwent exploratory laparotomy and bladder repair. During the procedure, systematic examination did not reveal any posterior urethral valves, anterior bladder perforations, or obstructive lesions. Posteriorly, a small, approximately 4 to 5 mm diameter, perforation along the posterior aspect of the bladder wall was identified and repaired. A Foley catheter was placed for postoperative drainage.

## 3. Discussion

### 3.1. Diagnostic Studies

Ultrasound, cystoscopy, and cystography have long been utilized as the imaging tools of choice for screening and visualization of bladder perforations with retrograde cystography, the gold standard recognized by the European Association of Urology.

### 3.2. Diagnostic Clues

Intraperitoneal rupture may present with increased intravesical pressure, abnormal serum electrolytes, and ascites. Urinary ascites has a unique biochemical profile and can be distinguished from other forms of ascites as reflected in Table 1. In theory, as urine builds up in the peritoneal cavity, creatinine, urea and potassium, driven by concentration gradients, are absorbed through the peritoneal lining into the bloodstream. Serum levels of urea nitrogen and creatinine are also uncharacteristically high. Serum electrolytes abnormalities reflecting hyperkalemia and hyponatremia have been reported in several case reports [2]. Other presenting factors of intra- and extraperitoneal ruptures will include hematuria, oliguria, lower abdominal pain, distention, ileus, peritonitis, or sepsis.

### 3.3. Management

The European Urological Association (EAU) and American Urological Associations (AUA) agree that the standard of care for intraperitoneal injuries is surgical exploration and repair, followed by postoperative drainage for 7–10 days. Exception to surgical exploration is made for the absence of peritonitis or ileus, where conservative management with a drain and antibiotics is acceptable. As evidence rises for the success of conservative management, more and more cases are being handled in this fashion and avoiding invasive surgical treatment. The AUA suggests performing a cystography after conservatively treated bladder injuries and surgically repaired extraperitoneal injuries, but follow-up imaging is not required after simple surgical repair of an intraperitoneal injury.

## 4. Conclusion

We postulate that our patient developed iatrogenic bladder perforation from the Foley catheter. This injury was likely an acute complication secondary to the ischemic damage to the bladder during his initial days of life when the patient was critically ill, ultimately requiring ECMO. Cell hypoxia leading to poor tissue integrity compromised the bladder, making it more susceptible to rupture. The diagnosis was established by the voiding cystourethrogram, which revealed intraperitoneal extravasation of contrast opacification of urinary bladder with features consistent with intraperitoneal bladder rupture. Our patient experienced fluctuating electrolyte derangements in sodium, potassium, BUN, and creatinine throughout the initial course of hospitalization. We speculate that the deranged electrolytes are secondary to the bladder rupture. The analysis of the peritoneal fluid revealed only a modest elevation of creatinine levels, 53 *μ*mol/L, as compared to the serum value, 39 *μ*mol/L. We postulate that peritoneal aspiration and Foley catheter placement prior to paracentesis may have impacted the creatinine levels. This also contributed to the delay in diagnosis. The presence of red blood cells of 493 cells/L suggests recent bladder rupture or continued blood loss secondary to injury of tissues and bladder wall vasculature.

Although our patient has had a smooth postoperative course, sequelae can be severe with permanent scarring of the bladder and poor emptying which can ultimately lead to bilateral hydronephrosis, renal damage, and chronic kidney failure.

## Figures and Tables

**Table 1:** Comparison of biochemical profile of the results of peritoneal taps reported in the literature.

Ascites	Protein	Color	Unique findings
Urinary [2, 3]	2–7 g/L	Yellow	High creatinine level (183 *µ*mol/L)^a^ High urea level (20 mmol/L)

Chylous [4]	2.5–4 g/dL	Milky^b^, straw^c^	High triglyceride (>110 mgr/dL), high chylomicrons, >500 cells/*µ*L (70–90% lymphocytes), bacteriostatic properties

Biliary [3, 5]	3.3 g/dL	Dark yellow-green	Bilirubin (30 mg/dL), WBC (1760/*µ*L, 90% neutrophils), sugar (80 mg/dL)

Our patient, day of life 25	—	Clear	Creatinine 53 *µ*mol/L, RBC 493/L, WBC 136/L, triglycerides 32 mg/dL, LDH 276 U/L

^a^Average of two patients; ^b^patient is orally fed; ^c^if patient is not orally fed.
